# Where is tibial edema located in cases of osteomeniscal impingement?

**DOI:** 10.1590/0100-3984.2022.0115

**Published:** 2023

**Authors:** Camilo Partezani Helito, Livia Dau Videira, Henrique Augusto Lino, Paulo Victor Partezani Helito, Marcelo Bordalo-Rodrigues

**Affiliations:** 1 Institute of Orthopedics and Traumatology, Hospital das Clínicas da Faculdade de Medicina da Universidade de São Paulo (HC-FMUSP), São Paulo, SP, Brazil; 2 Hospital Sírio-Libanês, São Paulo, SP, Brazil; 3 Aspetar Orthopaedic and Sports Medicine Hospital, Ad Dawhah, Doha, Qatar

**Keywords:** Meniscus, Knee joint, Magnetic resonance imaging, Menisco, Articulação do joelho, Ressonância magnética

## Abstract

**Objective:**

To characterize the location of tibial edema related to meniscal degeneration with a flap displaced into the meniscotibial recess (osteomeniscal impingement) on magnetic resonance imaging (MRI).

**Materials and Methods:**

We evaluated 40 MRI examinations of patients submitted to surgery due to inferior displacement of a meniscal flap tear into the meniscotibial recess and peripheral bone edema. Tibial edema was quantified in the coronal and axial planes.

**Results:**

On coronal MRI sequences, edema started in the tibial periphery and extended for a mean of 5.6 ± 1.4 mm, or 7.4 ± 2.1% of the tibial plateau. In the craniocaudal direction, the mean extension was 8.8 ± 2.9 mm. The mean ratio between the extent of craniocaudal and mediolateral edema was 1.6 ± 0.6. In the axial plane, the edema started in the medial periphery and extended for a mean of 6.2 ± 2.0 mm, or 8.2 ± 2.9% of the tibial plateau. In the anteroposterior measurement, the mean start and end of the edema was 21.4 ± 5.4 mm and 35.7 ± 5.7 mm, respectively, or 43.4 ± 10.2% and 72.8 ± 11.1% of the tibial plateau.

**Conclusion:**

Apparently, tibial edema resulting from osteomeniscal impingement always starts in the periphery of the meniscus. In the coronal plane, it appears to be more extensive in the craniocaudal direction than in the mediolateral direction. In the axial plane, we found it to extend, on average, approximately 6.2 mm in the mediolateral direction and to be most commonly located from the center to the posterior region of the medial tibial plateau.

## Abstract (pt)

**Objetivo:**

Caracterizar a localização do edema ósseo tibial relacionado a lesão meniscal degenerativa com fragmento deslocado no recesso meniscotibial (impacto osteomeniscal) por meio de ressonância magnética (RM).

**Materiais e Métodos:**

Quarenta RMs de pacientes submetidos a cirurgia por fragmento deslocado do menisco medial no recesso meniscotibial e edema ósseo periférico foram avaliadas. Edema ósseo tibial foi quantificado nos planos coronal e axial.

**Resultados:**

No plano coronal, o edema iniciou-se na periferia tibial e estendeu-se por 5,6 ± 1,4 mm, ou 7,4 ± 2,1% do platô. Na direção craniocaudal, o edema estendeu-se em média 8,8 ± 2,9 mm. A média entre a extensão do edema craniocaudal e mediolateral foi 1,6 ± 0,6. No plano axial, o edema iniciou-se na periferia medial e estendeu-se por 6,2 ± 2,0 mm, ou 8,2 ± 2,9% da medida da tíbia. Na medida anteroposterior, o edema iniciou-se em 21,4 ± 5,4 mm e terminou em 35,7 ± 5,7 mm ou iniciou-se em 43,4 ± 10,2% e terminou em 72,8 ± 11,1% do platô tibial.

**Conclusão:**

O edema ósseo tibial relacionado aos casos de impacto osteomeniscal sempre se inicia na periferia do menisco. Ele é mais extenso na direção craniocaudal do que mediolateral no plano coronal. No plano axial, ele estende-se por 6,2 mm de medial a lateral e é mais frequentemente localizado no centro da região posterior do platô medial.

## INTRODUCTION

Treatment of degenerative meniscal lesions is controversial^(1,2)^. Degenerative meniscal tears are characterized as lesions with a gradual onset of activity-related pain, unassociated with any major traumatic event. Degenerative lesions may progress poorly after surgical treatment because other, existing, degenerative changes can generate overload and pain. One of the signs of overload and degeneration is bone edema^(3,4)^, usually identified as a hyperintense signal on fluid-sensitive magnetic resonance imaging (MRI) sequences.

Chang et al.^(5)^ employed the term “shiny corners” to describe lesions with increased signal intensity in the tibial periphery on T2-weighted sequences. That finding may be associated with tears in the meniscal body or root. Okazaki et al.^(6)^ also studied “shiny corners” and concluded that they might be related to meniscal root tears when they are located more posteriorly. Krych et al.^(7)^ evaluated tibial edema in cases of meniscal lesion and concluded that the edema is more peripheral and localized when it is related to a displaced meniscal flap than when it is related to a meniscal root tear. However, those authors did not determine the exact location of the edema. In the preoperative evaluation, it is important to describe the location and extent of such edema because lesions associated with greater overload and degeneration tend to show less improvement after surgical treatment, although surgery can be successful if the edema is caused by a flap tear displaced into the meniscotibial recess^(8)^.

Displacement of a meniscal flap tear into the meniscotibial recess, resulting in bone edema, has had several names. The terms “osteomeniscal impingement”, “osteomeniscal impact”, “osteomeniscal impact edema”, and “meniscal comma sign” have all been used to characterize this pathology. Helito et al.^(8)^, Herschmiller et al.^(9)^, Krych et al.^(7)^, Lecas et al.^(10)^, and Jung et al.^(11)^ have suggested that surgical treatment produces good results for this type of lesion. To date, the associated edema has been characterized and localized only in a subjective manner, the observations having included only whether the edema is focal on the tibial periphery or diffuse throughout the tibial plateau, without objective parameters that could provide a more precise characterization. However, a meniscal flap tear is not always easy to characterize, because it can be mistaken for meniscal extrusion^(12)^. Therefore, there is a need for objective measures in order to improve its characterization.

The aim of this study was to determine the location of tibial edema related to inferior displacement of a meniscal flap tear, as well as to characterize the extent of that edema in the coronal and axial planes on MRI. We hypothesized that the edema would be in the tibial periphery, from the center to the anterior portion of the tibial plateau in the axial plane, and that its craniocaudal extent would be greater than its mediolateral extent in the coronal plane.

## MATERIALS AND METHODS

**Figure 1:**
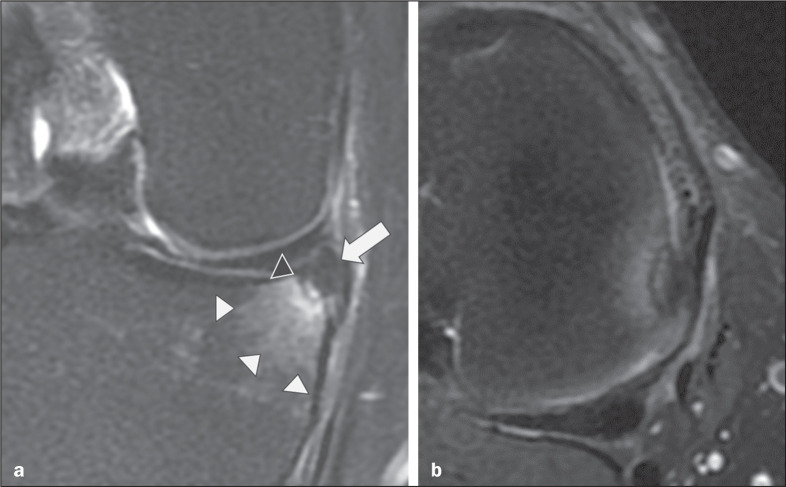
Typical findings of inferior displacement of a meniscal flap tear. T2-weighted MRI with fat saturation in the coronal and axial planes (a and b, respectively), showing a meniscal lesion with a flap (arrow) displaced into the meniscotibial recess, as well as an area of tibial edema (white arrowheads) next to the flap. In this case, adjacent bone remodeling (black arrowhead) can also be observed.

In this retrospective study, we evaluated the MRI scans of patients submitted to surgery due to inferior displacement of a meniscal flap tear into the meniscotibial recess and peripheral tibial edema (Figure 1). All examinations were performed at a single center between 2016 and 2021. All cases were confirmed by arthroscopy, and displacement of the meniscal flap was confirmed intraoperatively. Patients with meniscal root tears were excluded, as were those with Kellgren-Lawrence grade 2 or greater osteoarthritis (as determined by separate review of knee radiographs) and those with a history of surgery involving the affected knee. The study was approved by the institutional review board. Because of the retrospective nature of the study, the requirement for written informed consent was waived.

All images were acquired in 1.5 T scanners-Aera (Siemens Medical Solutions, Erlangen, Germany); Espree (Siemens Medical Solutions); Avanto (Siemens Medical Solutions); or Optima 450w (GE Healthcare, Milwaukee, WI, USA)-with dedicated knee coils. The parameters employed are described in Table 1.

**Table 1:** Parameters of the knee MRI protocol of the institution.

Sequence Parameter	TE (ms)	TR (ms)	FOV (cm)	Interslice gap (cm)	Slice thickness (cm)
Sagittal PDW	30-40	2,150-2,900	15-17	0.3-0.4	3.0-3.5
Coronal T1W	9-12	340-740	15-17	0.3-0.4	3.0-3.5
Axial T2W FATSAT	38-45	2,900-5,000	15-16	0.3-0.4	3.0-3.5
Sagittal T2W FATSAT	40-50	2,900-5,900	15-17	0.3-0.4	3.0-3.5
Coronal T2W FATSAT	40-50	2,200-4,500	15-17	0.3-0.4	3.0-3.5

TE, echo time; TR, repetition time; FOV, field of view; PDW, proton density weighted; T1W, T1-weighted; T2W, T2-weighted; FATSAT, fat-saturated.

On MRI, tibial edema was quantified in the coronal and axial planes. The area of edema was identified, and the slices in which the edema was more extensive were selected for measurement. In the coronal plane, the mediolateral length of the tibial plateau and the largest craniocaudal and mediolateral dimensions of the bone edema area were measured. The length ratio between the extent of bone edema in the mediolateral axis and that in the craniocaudal axis was also determined.

**Figure 2:**
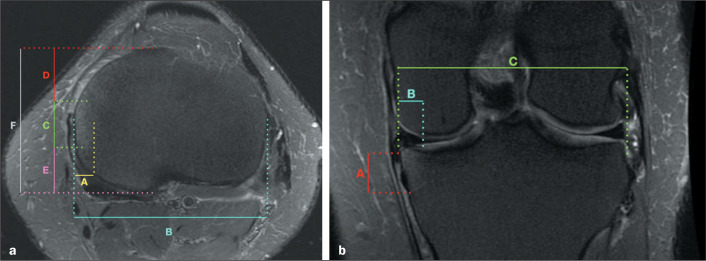
T2-weighted MRI with fat saturation, showing an example of bone edema measurements. a: In the axial plane, measurements were made of the largest mediolateral diameter of the edema (A) and tibia (B), together with the anteroposterior measurements of the edema (C), the distance from the edema to the most anterior cortex of the tibia (D), the distance from the edema to the most posterior cortex of the tibia (E), and the greatest distance between the anterior and posterior cortex of the tibia (F). b: In the coronal plane, craniocaudal and mediolateral measurements of the edema were made (A and B, respectively), as was a measurement of the mediolateral length of the tibial plateau (C).

In the axial plane, the greatest distances between the anterior and posterior cortex and between the medial and lateral cortex of the tibia were measured, as were the largest anteroposterior and mediolateral dimensions of the bone edema area, as well as the distances between the anterior margin of the edema area and the anterior cortex of the tibia and between the posterior margin of the edema area and the posterior cortex of the tibia. The way in which the measurements were made is shown in Figure 2.

A knee surgeon and a musculoskeletal radiologist made each measurement twice, with an interval of at least 30 days between the two measurements, and correlation measurements were also made. Each evaluator was instructed to draw all lines at the time of the measurements.

The results were analyzed by using descriptive statistics, including mean, minimum, maximum, and standard deviation. In addition, correlations between the values obtained by the two evaluators were analyzed by calculating the intraclass correlation coefficient. The initial measurements of the more experienced evaluator (the knee surgeon) were used for the analysis, the other measurements being used only for the correlation tests.

## RESULTS

We evaluated 40 MRI scans from 40 patients (14 women and 26 men). The mean age of the patients was 61.2 ± 12.2 years. Of the 40 scans evaluated, 18 were of the right leg and 22 were of the left leg.

**Figure 3:**
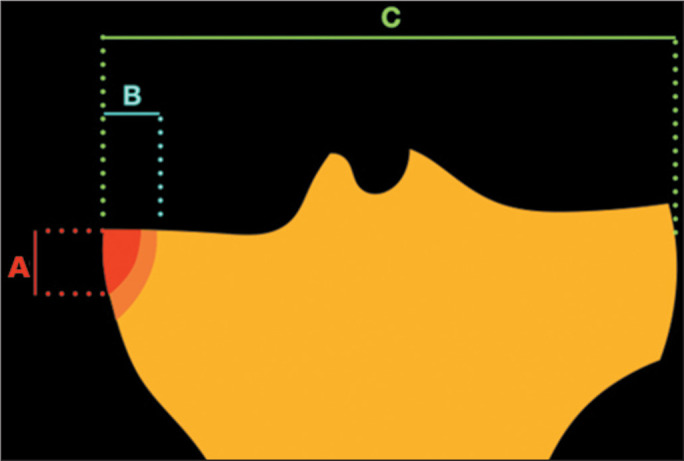
Schematic drawing showing the location of tibial edema in cases of inferior displacement of a meniscal flap tear in the coronal plane. The red area (A) represents the means of the measurements-5.46 mm in the mediolateral direction and 8.88 mm in the craniocaudal direction; and the orange area (B) represents the standard deviation-1.4 mm for the mediolateral direction and 2.9 mm for the craniocaudal direction. The mean mediolateral measurement of the tibia was 75.9 ± 7.0 mm and is represented by the letter C.

In the coronal plane, the mean length of the tibia was 75.9 ± 7.0 mm. In all cases, tibial edema started in the periphery of the bone and extended for a mean of 5.6 ± 1.4 mm, or 7.4 ± 2.1% of the tibial plateau. In the craniocaudal direction, the edema extended for a mean of 8.8 ± 2.9 mm. The mean ratio between the craniocaudal and mediolateral extent of edema was 1.6 ± 0.6 (Figure 3). In 29 (72.5%) of the cases in our sample, the edema started below the middle of the medial plateau.

**Figure 4:**
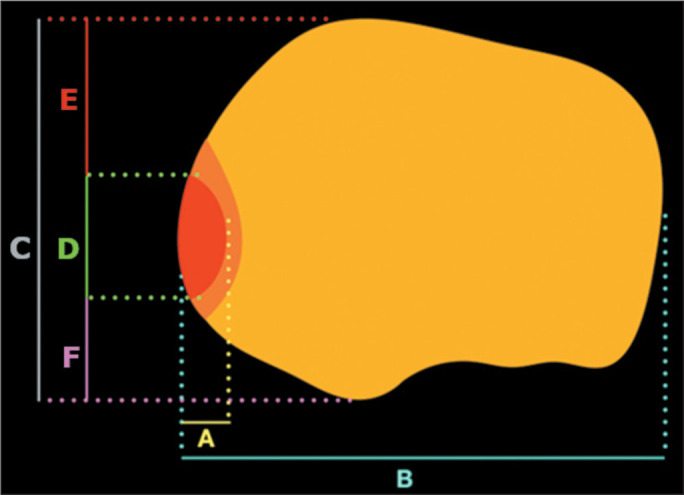
Schematic drawing showing the location of tibial edema in the axial plane in cases of inferior displacement of a meniscal flap tear. The red area represents the mean location of the edema, the orange area including the standard deviation. The letter A indicates the mean mediolateral measurement of the edema (6.2 ± 2.0 mm), and the letter B indicates the mean mediolateral measurement of the tibia (77.2 ± 7.1 mm). In the anteroposterior direction, the letter F represents the mean anteroposterior measurement of the tibia (49.4 ± 5.5 mm), the letter C represents the overall extent of the edema, the letter D represents the measurement from the beginning of the tibia to the beginning of the edema, and the letter E represents the measurement from the end of the edema to the end of the tibia.

Table 2 shows the measurements obtained in the axial and coronal planes. In the axial plane, the mean length of the tibia in the anteroposterior and mediolateral directions was 49.4 ± 5.5 mm and 77.2 ± 7.1 mm, respectively. In all cases, the edema started in the medial periphery and extended for a mean of 6.2 ± 2.0 mm, or 8.2 ± 2.9% of the mediolateral length of the tibia. In the anteroposterior direction, the edema, measured from the most anterior part of the tibia, started and ended, respectively, at 21.4 ± 5.4 mm and 35.7 ± 5.7 mm, or 43.4 ± 10.2% and 72.8 ± 11.1% of the anteroposterior length of the tibia (Figure 4). For the variables studied, the intraclass correlation coefficient for interand intra-observer agreement ranged from 0.81 to 0.91 and from 0.82 to 0.93, respectively.

**Table 2:** Axial and coronal MRI measurements in cases of inferior displacement of a meniscal flap tear.

Measurement	Mean ± SD (range)
Length of the tibia in the coronal plane (mm)	75.9 ± 7.0 (60.4-85.3)
Mediolateral extent of edema in the coronal plane (mm)	5.6 ± 1.4 (2.2-7.9)
Mediolateral extent of edema in the coronal plane (%)	7.4 ± 2.1 (2.9-12.0)
Craniocaudal extent of edema in the coro-nal plane (mm)	8.8 ± 2.9 (4.9-18.0)
Craniocaudal and mediolateral measure-ment of edema in the coronal plane	1.6 ± 0.6 (1.0-3.7)
Anteroposterior length of the tibia in the axial plane (mm)	49.4 ± 5.5 (41.3-58.4)
Start of anteroposterior edema in the axial plane (mm)	21.4 ± 5.4 (13.1-30.7)
Start of anteroposterior edema in the axial plane (%)	43.4 ± 10.2 (28.3-63.4)
End of anteroposterior edema in the axial plane (mm)	35.7 ± 5.7 (27.0-46.3)
End of anteroposterior edema in the axial plane (%)	72.8 ± 11.1 (53.0-92.3)
Anteroposterior extent of edema in the axial plane (mm)	14.3 ± 4.5 (5.9-23.4)
Mediolateral length of the tibia in the axial plane (mm)	77.2 ± 7.1 (60.6-86.7)
Start of mediolateral edema in the axial plane (mm)	^*^
End of mediolateral edema in the axial plane (mm)	6.2 ± 2.0 (2.4-11.1)
End of mediolateral edema in the axial plane (%)	8.2 ± 2.9 (3.1-17.1)
Mediolateral extent of edema in the axial plane (mm)	6.2 ± 2.0 (2.4-11.1)

* Always at the edge of the medial tibial plateau.

## DISCUSSION

The main achievement of this study is the detailed topographic description of tibial bone edema in cases of inferior displacement of a meniscal flap tear. Our results indicate that the hyperintense signal (edema) observed on fluid-sensitive MRI sequences acquired in the coronal plane is significantly more pronounced in the craniocaudal direction than in the mediolateral direction. In the axial plane, we found the edema to extend approximately 14 mm from anterior to posterior and to occupy an area extending from the center to the posterior region of the medial tibial plateau. Thus, we have partially confirmed our hypothesis. In the coronal plane, the extent of tibial edema was greater in the craniocaudal direction, as we had hypothesized. However, we had also hypothesized that, in the axial plane, the edema would extend from the center to the anterior region of the medial tibial plateau, rather than to its posterior region.

In several studies that focused on evaluating degenerative meniscal lesions, the authors concluded that surgery provides no benefit to the patient and that the treatment should therefore be conservative^(13,14)^. Each of those studies evaluated a very broad spectrum of patients collectively, which generates significant bias because degenerative meniscal lesions can present in different ways^(15-18)^.

One example of a degenerative lesion for which conservative treatment produces poor results and good results with surgical treatment provides good results is a meniscal root tear^(19,20)^. Recent studies have shown that surgical repair of a meniscal root tear can, in addition to improving the clinical status of patients, delay the progression of osteoarthrosis. Degenerative lesions with flaps displaced into the meniscotibial recess may also benefit from surgical treatment. Helito et al.^(8)^ showed that only approximately 20% of patients with such lesions improved with conservative treatment, the remaining patients being submitted to surgical treatment. The challenge in this type of lesion is to determine whether the edema is due to the displacement of the meniscal flap or to a more global overload in the compartment, which might not improve with surgical treatment^(21)^. There is a broad spectrum of pathologies that can lead to signal changes around the knee joint^(22)^. As mentioned in previous studies on this topic^(6-8,10)^, a displaced meniscal flap is often mistaken for a simple meniscus extrusion; it is therefore important to characterize the resulting edema in order to distinguish between the two.

Perhaps the most significant finding of this study is that, in the coronal plane, the extent of edema was greater in the craniocaudal direction than in the mediolateral direction. That suggests that the edema is caused not only by increased load and pressure on the joint but also by the pressure of the flap in the meniscotibial recess. When this relationship between the craniocaudal and mediolateral measurements of edema is observed, there is a high probability that it is a case of inferior displacement of a meniscal flap tear. In our sample, there were no cases in which the extent of edema was greater in the mediolateral direction than in the craniocaudal direction, and the two measurements were similar (with a ratio of 1.0) in only one case.

In the present study, the location of the edema in the axial plane was also peripheral, extending, on average, only 6 mm in the mediolateral direction and always starting in the most peripheral portion of the tibial plateau. That finding is significant because edema starting further from the periphery is probably unrelated to inferior displacement of a meniscal flap tear. In the anteroposterior direction, the edema occupied an area from the center to posterior, contradicting our initial hypothesis, which was based on previous intraoperative observations suggesting that the location would be more anterior. In most (72.5%) of the cases in our sample, the edema started below the middle of the medial plateau. In all cases, the edema ended after halfway through the plateau.

The characterization of tibial edema related to cases of inferior displacement of a meniscal flap tear is another practical tool for the correct diagnosis of this pathology and the possible indication of surgical treatment for flap resection and pain reduction. Helito et al.^(8)^ showed that the postoperative evolution after flap resection was unsatisfactory only in smokers and patients with varus alignment. It is still unknown whether flap resection leads to the reduction or resolution of tibial edema. Although that was suggested by Helito et al., there is still no evidence in that regard.

This study has some limitations. Only a relatively small number of cases were evaluated, and the measurements were performed manually by the evaluators through image assessment platforms. Nevertheless, we believe that 40 cases were sufficient for this characterization. We also believe that the measurements were performed correctly, given the good intraclass correlation coefficient values. Another limitation is the absence of functional scales to evaluate a possible correlation between the extent of the edema and the pain symptomatology. However, that would go beyond the initial study question, which was about the location of such edema. Other conditions responsible for subchondral bone marrow edema with a diffuse or central presentation, such as insufficiency fractures or degenerative changes, have not being studied in the same way for comparison, therefore requiring future studies for evaluation. A final limitation is the fact that the patients did not undergo postoperative MRI to evaluate the possible reduction in bone edema.

In conclusion, the tibial edema related to cases of inferior displacement of a meniscal flap tear appears to always start in the periphery of the meniscus. In the coronal plane, such edema is apparently more extensive in the craniocaudal direction than in the mediolateral direction. Our findings indicate that, in the axial plane, it extends approximately 6.2 mm in the mediolateral direction and is most frequently located from the center to the posterior region of the medial tibial plateau.
